# Comment on: The perfect stoma: tips from a stoma nurse

**DOI:** 10.1093/bjs/znae011

**Published:** 2024-01-25

**Authors:** Francesco Pata, Dario Parini, Gianluca Rizzo, Andrea Bondurri, Francesco Ferrara

**Affiliations:** Department of Pharmacy, Health and Nutritional Sciences, University of Calabria, Cosenza, Italy; General Surgery Department, Santa Maria della Misericordia Hospital, Rovigo, Italy; Digestive and Colorectal Unit, Fatebenefratelli Isola Tiberina Gemelli Isola Hospital, Università Cattolica del Sacro Cuore, Rome, Italy; General Surgery Department, Luigi Sacco University Hospital, ASST Fatebenefratelli-Sacco, Milan, Italy; Department of Surgical, Oncological and Stomatological Sciences (DICHIRONS), University of Palermo, Palermo, Italy; Unit of General and Oncological Surgery, ‘Paolo Giaccone’ University Hospital, Palermo, Italy

*Dear Editor*

The article by Krogsgaard *et al*.^1^ is interesting and provides an insightful perspective on optimizing outcomes for ostomy patients.

It is agreed that preoperative planning and postoperative care are crucial to achieve optimal results in stoma formation, especially by involving stoma care nurses at an early stage. However, there are certain assertions that call for further scrutiny based on existing literature and guidelines.

The routine use of a rod or bridge in loop ostomy has been found to be ineffective in lowering the rate of stoma retraction and increases the likelihood of some complications (including necrosis, infection, or dermatitis). Therefore, the evidence available suggests avoidance of the regular use of a rod in loop ostomy formation.

There is no existing evidence to suggest the superiority of transrectal location over the pararectal approach for the prevention of parastomal hernia. Furthermore, there is no evidence about the caudal placement of the oral end in loop ostomies to minimize the risk of faecal overflow.

The suggestion to use prophylactic mesh in permanent end colostomies to reduce the risk of parastomal hernia is well taken. However, in the light of recent evidence, it should be balanced against the risk of potential mesh-related complications, such as infection or erosion.

It is agreed that the protrusion of any colostomy or ileostomy above the skin surface is key to reducing postoperative complications.

A prompt stoma reversal/closure, whenever possible, should be included in mitigation strategies to reduce stoma complications and improve the quality of life for patients who have ostomy creation.

As a multidisciplinary group, the Multidisciplinary Italian Study group for STOmas (‘MISSTO’) advocates for increased collaborative decision-making between surgeons, stoma care nurses, and patients. This approach could mitigate the silo mentality and foster more effective patient care strategies.
